# The interaction between litter input and soil microbial community regulates the intraspecific allelopathic effects of *Solanum rostratum* Dunal

**DOI:** 10.3389/fpls.2026.1769927

**Published:** 2026-02-27

**Authors:** Huixian Liu, Yujuan Zhang, Juan Qiu, Shuai Liu, Nusratgul Anwar, Lamei Jiang, Fang Wang, Jing Deng, Shanshan Wang, Dunyan Tan

**Affiliations:** 1 Xinjiang Key Laboratory for Ecological Adaptation and Evolution of Extreme Environment Organisms, College of Life Sciences, Xinjiang Agricultural University, Ürümqi, China; 2 Halliburton, Perth, WA, Australia; 3 College of Chemistry, Xinjiang University, Urumqi, China; 4 State Key Laboratory of Ecological Safety and Sustainable Development in Arid Lands, Xinjiang Institute of Ecology and Geography, Chinese Academy of Sciences, Ürümqi, China

**Keywords:** allelopathy, litter, soil metabolites, soil microorganisms, *Solanum rostratum* Dunal

## Abstract

**Introduction:**

The "novel weapon hypothesis" posits that invasive plants suppress native species by releasing allelochemicals, which is a crucial factor for their successful invasion. While most studies focus on interspecific allelopathy, with insufficient attention paid to intraspecific allelopathy.

**Methods:**

This study employed an in pot experiment with different litter concentrations (0, 5, 10, 20 g/kg) to cultivate *Solanum rostratum* Dunal seedlings in both sterilized and unsterilized soils. The plant growth parameters, soil physicochemical properties, soil metabolites, and soil microbial communities were measured, and their interrelationships were also analyzed.

**Results:**

The results indicated that the litter from *Solanum rostratum* Dunal significantly inhibited the growth of its seedlings, and the inhibitory effect is even stronger in sterile soil. Additionally, the addition of litter decreased the soil pH value, while increasing the soil electrical conductivity, total carbon, total nitrogen and total phosphorus contents. Metabolomic analysis identified the phenolic compound 4-Ethyl-2-methylphenol and the ester compound Carvyl propionate as main secondary metabolites in soil, whose concentrations showed significant negative correlations with seedling growth. In unsterilized soil, the abundance of *Sphingomonas* and *Dongia*—bacteria with degradation potential—increased, exhibiting negative correlations with allelopathic metabolite levels and positive correlations with seedling growth indicators.

**Discussion:**

In summary, the self toxic effect of Solanum rostratum Dunal litter on seedling growth increased with the increasing of litter content, and soil microorganisms mitigate the allelopathic effects by degrading or transforming allelopathic compounds in litter, thereby playing a crucial role in regulating its invasion process.

## 1 Introduction

The new weapon hypothesis suggests that an important reason for the success of invasive plant species is their ability to produce certain allelochemicals. These allelochemicals are released into the environment to inhibit the growth of local plants. While the native plants in the invaded area have no co-evolution history with the invasive species, and cannot adapt to the specific allelochemicals produced by the invasive plants, thus showing sensitivity to the invasive plants (Macel et al., 2014). The litter of invasive plants, as a natural carrier of allelochemicals that are continuously input, are believed to release secondary metabolites during decomposition, which play a continuous and crucial ecological role in the invasion process. Studies have shown that the litter of invasive plants can directly inhibit the germination of local plant seeds, the growth of seedlings, and the development of root systems by releasing allelochemicals such as phenolic acids, terpenoids, and alkaloids, thereby weakening interspecific competition and promoting the expansion of their own populations (Heuermann et al., 2023). For instance, the leaf filtrate of the invasive plant *Mikania micrantha* increased the water-soluble phenolic compounds in the soil, thereby inhibiting the growth of other plants (Kaur et al., 2012).

On the other hand, the chemical substances produced by invasive alien plants can act on the microorganisms in the soil, altering their population numbers, community structure and functions, disrupting the interactions and symbiotic relationships between local plants and soil microorganisms, and facilitating the success of their invasion (Kong et al., 2017). For instance, chemicals produced by invasive plants can act on soil microorganisms, altering their population abundance, community structure, and function. This disrupts native plant-soil microorganism interactions and symbiotic relationships, thereby advancing their invasion success (Kong et al., 2017). However, the competitive advantage of invasive species over native species is often short-lived and only important in the early stages of the invasion process. The competitive ability of invasive species may decline over time (Gioria and Osborne, 2014). For example, in soils with longer invasion histories, invasive species exhibit lower survival rates, biomass, and competitive ability (Mitchell et al., 2011; Dostál et al., 2013). The allelopathic effect is not a constant negative effect, but a dynamic process regulated by environmental factors. allelopathic substances not only inhibit local plants, but may also have inhibitory effects on invaders themselves, known as “Autotoxicity”. This may be a mechanism of population self-regulation, and its ecological consequences need to be evaluated on a more complex spatiotemporal scale. Autotoxicity is one of the important forms of allelopathy, and it is a form of chemical communication between organisms. It refers to the phenomenon where plants release chemical substances through aboveground parts, root exudation, gas volatilization and decomposition of plant residues into the environment, inhibiting the growth of the same species of plants. Studies have found that plants can change the soil microbial community, thereby determining the growth and defense of the next generation. This is relatively common in agricultural ecosystems. For example, the allelochemicals released by corn can change the composition of bacterial and fungal communities in the soil, and these changes increase the defense ability of leaves and reduce the growth and yield of plants (Hu et al., 2018). On the other hand, soil microorganisms have the ability to degrade the allelochemicals of invasive plants, which can alleviate the allelopathic effect during the process of biological invasion (Li et al., 2015). Most studies have focused on the immediate effects of root exudation in living plants or leaching from the above-ground parts (Yu et al., 2022; Sun et al., 2022), while generally neglecting the important ecological process of litter decomposition, which continues over time and accumulates in space. It is not yet clear how the accumulation of litter, as a “reservoir” and slow release source of allelopathic substances, modulates the intensity of self toxicity.

The invasive plant *Solanum rostratum* Dunal is classified as a highly dangerous quarantine plant (Zhao et al., 2019). It is spiny all over and poisonous, which can reduce livestock fur yield and increases the risk of spreading crop pests and diseases. Its unique seed structure enables prolonged viability under harsh conditions with minimal soil requirements. Furthermore, extensive research indicates that *Solanum rostratum* Dunal exhibits allelopathic effects, completely inhibiting the germination of *Amaranthus retroflexus* seeds while suppressing those of *Poa annua* and *Medicago sativa* by 55.25% and 51.59%, respectively (Zhou et al., 2021). *Solanum rostratum* Dunal exhibits strong overall adaptability and rapid growth. Following invasion, it often forms extensive monoculture stands (**Supplementary Figure 1**). Upon plant maturity, leaf senescence and stem breakage lead to plant material entering the soil, readily inducing intraspecific allelopathy (Wang et al., 2021). However, research on intraspecific allelopathy in *Solanum rostratum* Dunal remains limited, and its role in interactions with soil microorganisms during the *Solanum rostratum* Dunal dispersal and spread remains incompletely understood.

This study utilized the invasive plant *Solanum rostratum* Dunal as research material. Through controlled laboratory experiments, we measured the characteristics of *Solanum rostratum* Dunal seedling growth in soils treated with different litter applications (sterilized and unsterilized soils). combined with microbiome and metabolomics technologies, to reveal the role of soil microorganisms in the intraspecific allelopathy mediated by *Solanum rostratum* Dunal litter. The findings provide theoretical support from a microbial and intraspecific allelopathy perspective for understanding the invasion mechanisms of *Solanum rostratum* Dunal.

## 2 Materials and methods

### 2.1 Collection and preservation of *Solanum rostratum Dunal* litter and soil

The *Solanum rostratum* Dunal community in Shirengou, Shuimogou District, Urumqi City (43.82864138 N, 87.78852892 E) was selected, and the whole *Solanum rostratum* Dunal litter was collected and dried at room temperature, ground through a 60-mesh sieve, and put into the polyethylene bag and stored at room temperature. The soil was collected from this community (where the soil was free from *Solanum rostratum* Dunal), the surface vegetation and debris were removed, and collected soil from a depth of 10 cm below the surface, brought back to the laboratory, dried at room temperature, crushed through 10-mesh sieve, and bagged it for future use (Li et al., 2015).

### 2.2 Potting experiments and physiological indexes of *Solanum rostratum* Dunal

*Solanum rostratum* Dunal litter and habitat soil were respectively mixed and placed in pots at a concentration gradient of 0, 5, 10, and 20 g. Each pot contained 1 kilogram of soil and different amounts of litter. Set up both sterilized and non-sterilized treatments. Sterilized soil was prepared by autoclaving at 121 °C for one hour, with a 24-hour interval between each of the three sterilization cycles (Li et al., 2015; Su et al., 2019). Selected *Solanum rostratum* Dunal seeds of uniform size that had broken dormancy and sowed 10 seeds per pot, covered with 1 cm of soil, and three replicates were set up for each concentration. All treatments were placed in the 12/12 h light/dark cycle greenhouse culture with 350 μmol/m^2^/s photosynthetically active radiation at 30 °C/20 °C and watered every 3 days. Continuous cultivation for 45 days. The germination rate, plant height, root length and dry weight of the plants were determined under different treatments, and the total carbon, organic carbon, total nitrogen, ammoniacal nitrogen, total phosphorus, pH and conductivity of the soil were measured.

### 2.3 Pseudotargeted metabolomics detection of soil

All soil samples underwent sequential homogenization, ultrasonication, sedimentation, and centrifugation. They were then filtered through 0.22 μm membranes into detection vials for subsequent analysis. LC-MS analysis was performed using a Thermo Vanquish (Thermo Fisher Scientific, USA) ultra-high-performance liquid chromatography system. Mass spectrometry employed dual-mode ionization scanning (positive and negative ions). Positive ion spray voltage was set at 3.50 kV, negative ion spray voltage at -2.50 kV, with a sheath gas flow of 40 arb and an auxiliary gas flow of 10 arb. The capillary temperature was maintained at 325 °C. First-stage and second-stage full-scan resolutions were 60000 and 15000, respectively.

### 2.4 High-throughput sequencing of soil

Soil microbial communities of *Solanum rostratum* Dunal were analyzed via high-throughput sequencing. Total DNA from the soil microbial community was extracted using the E.Z.N.A.^®^ Soil DNA Kit (Omega Bio-tek, Norcross, GA, U.S.). Concentration and purity were determined using NanoDrop 2000, and DNA extraction quality was assessed via 1% agarose gel electrophoresis. The bacterial community 16S rRNA gene was amplified using primers 338F (5’-ACTCCTACGGGAGGCAGCAG-3’) and 806R (5’-GGACTACHVGG GTWTCTAAT-3’). The fungal Internally Transcribed Spacer was amplified using primers ITS1F (5’ -CTTGGTCATTTAGAGGAAGTAA-3’) and ITS2R (5’-GCTGCGTTCTTCATCGATGC-3’). Detect amplification quality via 2% agarose gel electrophoresis. Quantify using Qubit 4.0 (Thermo Fisher, USA), then perform PE300 sequencing on the Illumina MiSeq platform.

Sequencing reads were demultiplexed, quality controlled by fastp (version 0.21.0), and merged by FLASH (version 1.2.7). Shortly, reads with adaptor sequences and low quality bases (quality score <Q20) were trimmed. Truncated reads shorter than 50 bp and reads containing ambiguous nucleotides were discarded. Subsequently, the paired-end reads were merged according to the minimum overlap of 10 bp with maximum mismatch ratio of 0.2 in the overlapping region. Only merged sequences were retained for downstream analyses.

### 2.5 Metabolomics and high-throughput sequencing data analysis

Peak detection, peak filtering and peak alignment were processed using the R XCMS software package to obtain the quantitative list of substances. Inter-sample response intensity variations were corrected using sum normalization, and variables with QC sample RSD > 30% were excluded to eliminate systematic errors. Multivariate statistical analysis was performed using the Ropls package in R. Metabolites showing significant differences underwent pathway enrichment analysis via the KEGG database.

Sequences were operational taxonomic unit (OTU) clustered using Vsearch based on 97% similarity to obtain OTUs and a feature list. The OTU sequences were taxonomically annotated to species using the RDP classifier, a ribosomal database project, and community composition was statistically analyzed for each sample at various taxonomic levels. Perform statistical analysis and data visualization using R 4.3.1.

The R (version 4.1.3) was used to perform general statistical analysis and visualize results via packages vegan (v2.6-4), phyloseq (v1.38.0), tidyverse (v1.3.2), ggpubr (v0.5.0), ComplexHeatmap (v2.10.0) and corrplot (v0.92). Alpha diversity was estimated using the ACE, Chao1 and Shannon indices. For pairwise comparisons in α diversity difference tests, the Wilcoxon test is employed. Principal coordinates analysis (PCoA) based on bray-curtis matrices with statistical significance determined by permutational multivariate analysis of variance (PERMANOVA) was conducted to assess the differences in beta diversity between groups. For comparing the relative abundance of different taxa between groups, linear discriminant analysis (LDA) effect size (LEfSe) method was performed with a p-value < 0.05 for the Kruskal-Wallis test. Spearman’s rank correlation analysis was used for correlation analysis.

### 2.6 Statistical analysis

The test of intergroup variability and one-way ANOVA was performed on the physiological indices of the plants using SPSS 20.0, and the results were significantly different by Duncan’s test (*P* < 0.05).

## 3 Results

### 3.1 Effects of different concentrations of *Solanum rostratum* Dunal litter on the growth of its own seedlings

**Figure 1:**
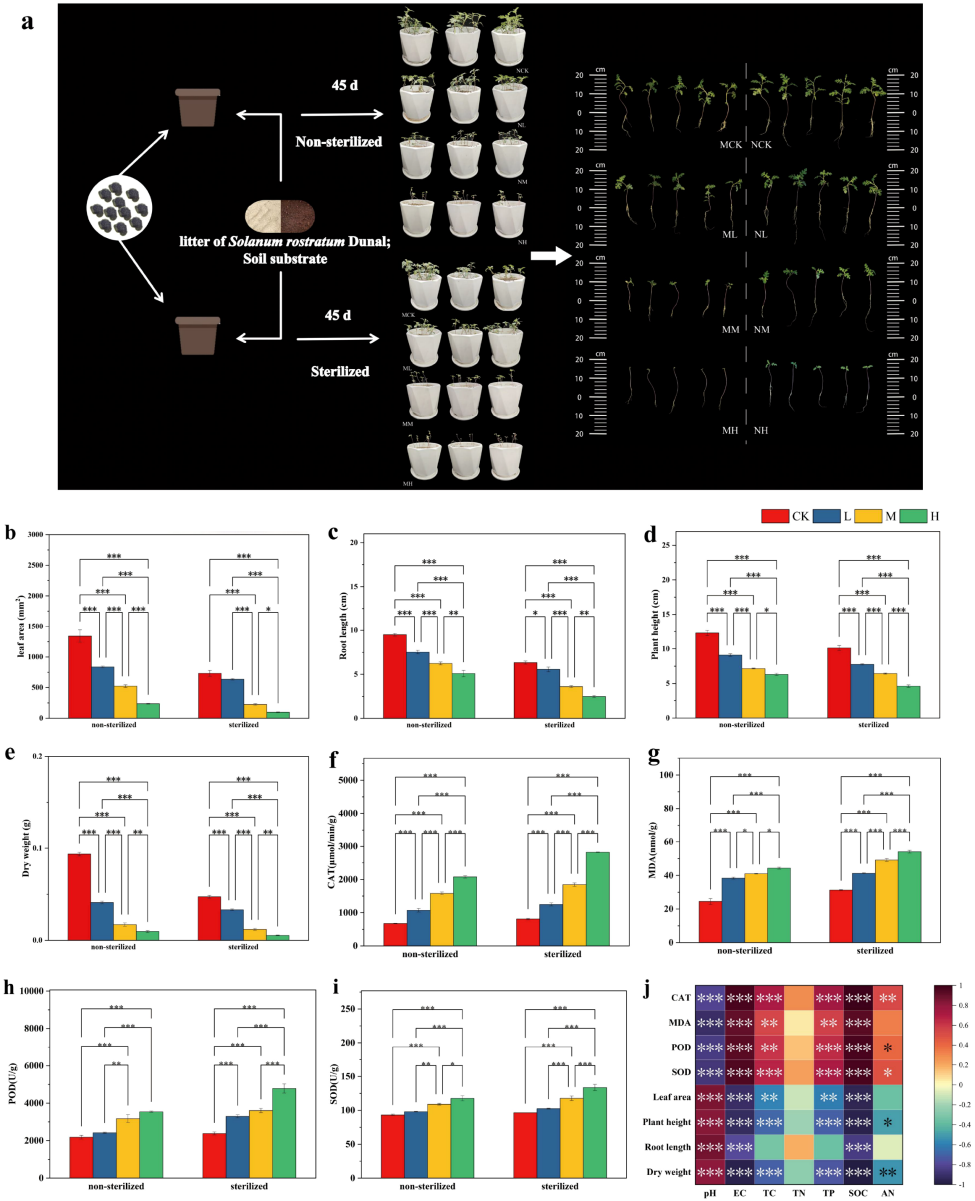
Effects of different concentrations of *Solanum rostratum* Dunal litter on various physiological indicators. (**(a)** Experimental design (MCK, ML, MM, MH represent sterile conditions: blank (no litter), low concentration (5g), medium concentration (10g), high concentration (20g); NCK, NL, NM, NH represent non-sterile conditions: blank, low concentration, medium concentration, high concentration); **(b)** Leaf area; **(c)** Root length; **(d)** Plant height; **(e)** Dry weight; **(f)** CAT; **(g)** MDA; **(h)** POD; **(i)** SOD; **(j)** Correlation between soil physicochemical properties and seedling physiology. *P < 0.05, **P < 0.01, ***P < 0.001. The number of pots used for statistical analysis is 3, with 10 seeds per pot).

The effects of different concentrations of *Solanum rostratum* Dunal litter on seedling growth are shown in **Figure 1**. Results indicate that as the concentration of *Solanum rostratum* Dunal litter increased, plant leaf area, plant height, root length, and biomass all decreased significantly, while CAT, MDA, POD, and SOD activities all increased significantly (**Figures 1b-i**). Under sterilized conditions, the inhibitory effect of litter was more pronounced. Compared to the CK, the addition of high-concentration litter under unsterilized conditions reduced plant leaf area, plant height, and root length by 82.36%, 48.72%, and 46.46%, respectively. while CAT, MDA, POD, and SOD activities increased by 66.95%, 42.95%, 38.32%, and 20.80%, respectively. Under sterilized conditions, plant leaf area, plant height, and root length decreased by 86.68%, 54.56%, and 60.89%, while CAT, MDA, POD, and SOD activities increased by 71.20%, 46.33%, 50.20%, and 27.93%, respectively. At the same litter concentration, sterilization status significantly affected seedling growth. Unsterilized soil can mitigate the inhibitory effect of *Solanum rostratum* Dunal litter on seedlings. Specifically, when high-concentration litter was added, compared to the sterilized treatment, the unsterilized treatment increased seedling leaf area, plant height, and root length by 58.80%, 26.98%, and 51.31%, respectively, while CAT, MDA, POD, and SOD activities decreased by 26.34%, 19.06%, 26.01%, and 11.93%.

Additionally, we measured the physicochemical parameters of the soil under different treatments (**Table 1**). Results indicate that under both sterilized and unsterilized conditions, soil pH gradually decreased with increasing amounts of *Solanum rostratum* Dunal litter, while soil electrical conductivity, total carbon, total nitrogen, total phosphorus, soil organic carbon, and available nitrogen showed increasing trends. Furthermore, under identical litter addition levels, sterilized treatments exhibited lower soil pH, total carbon, total nitrogen and total phosphorus compared to unsterilized treatments, while electrical conductivity and soil organic carbon were higher.

**Table 1:** Effects of the litter of *Solanum rostratum* Dunal on soil physical and chemical properties under sterilization and non-sterilization.

Group\Parameters	pH	EC(μs/cm)	TC(g/kg)	TN(g/kg)	TP(g/kg)	SOC(g/kg)	AN(mg/kg)
NCK	7.49± 0.07 Aa	567.33± 24.09 Aa	11.41± 0.09 Aa	0.99± 0.04 Aa	0.25± 0.005 Aa	6.94± 0.25 Aa	1.40± 0.05 Aa
NL	7.27± 0.03 Ba	630.00± 1.73 Ba	12.41± 0.29 Ba	1.14± 0.02 Aa	0.34± 0.003 Ba	7.70± 0.15 Ba	1.70± 0.05 Ba
NM	7.23± 0.04 Ba	719.33± 17.38 Ca	13.39± 0.52 Ca	1.52± 0.07 Ba	0.41± 0.007 Ca	8.79± 0.20 Ca	2.02± 0.10 Ca
NH	7.23± 0.03 Ba	773.67± 26.85 Da	14.51± 0.44 Da	1.87± 0.14 Ca	0.56± 0.012 Da	10.47± 0.27 Da	3.88± 0.07 Da
MCK	7.28± 0.03 Ab	581.33± 22.14 Aa	10.16± 0.53 Ab	0.49± 0.02 Ab	0.11± 0.006 Ab	7.44± 0.06 Ab	1.18± 0.07 Ab
ML	7.20± 0.02 Ba	660.00± 6.55 Bb	11.03± 0.04 Bb	0.53± 0.02 ABb	0.19± 0.019 Bb	8.13± 0.08 Ab	1.32± 0.02 Bb
MM	7.15± 0.01 Ca	725.00± 15.13 Ca	11.78± 0.31 Cb	0.58± 0.01 Bb	0.29± 0.008 Cb	9.68± 0.48 Bb	1.42± 0.02 Cb
MH	7.12± 0.01 Cb	773.67± 13.05 Da	13.33± 0.14 Db	0.76± 0.05 Cb	0.49± 0.006 Db	11.94± 0.57 Cb	1.60± 0.05 Db

(Data are presented as mean ± SD (n=3). Values in the same column followed by different letters indicate significant differences, Capital letters (A, B) indicate the significance of the differences between the addition of different litter under sterilized or unsterilized treatment; lowercase letters (a, b) indicate the significance of the difference between sterilized and non-sterilized at the same concentration. (*P* < 0.05, one-way ANOVA, Duncan’s test). EC, Electrical conductivity; TC, total carbon; TN, total nitrogen; TP, total phosphorus; SOC, Soil Organic carbon; AN, Ammonia nitrogen).

Correlation analysis was conducted between soil physicochemical properties under different treatments and seedling physiological indicators (**Figure 1j**). Results revealed that soil pH showed a significant positive correlation with plant leaf area, plant height, root length, and dry weight, while exhibiting a significant negative correlation with plant CAT, MDA, POD, and SOD activities. Soil electrical conductivity and soil organic carbon content showed significant negative correlations with plant height, root length, and dry weight, while exhibiting significant positive correlations with CAT, MDA, POD, and SOD activities. Soil total carbon and total phosphorus showed significant negative correlations with leaf area, plant height, and dry weight, and significant positive correlations with CAT, MDA, POD, and SOD activities. Soil available nitrogen content showed a significant negative correlation with plant height and dry weight, and a significant positive correlation with plant CAT, POD, and SOD activities.

### 3.2 Metabolites in unsterilized soil and sterilized soil under different treatment of litter

Identification of metabolites in sterilized and unsterilized soils revealed no differences in the types of metabolites present between the two groups, but significant variations in metabolite concentrations were observed. A total of 624 metabolites were isolated and identified from the soil, classified into 21 categories. The most abundant class was fatty acyls (73, 11.70%), followed by organooxygen compounds (46, 7.37%), prenol lipids (45, 7.21%), carboxylic acids and derivatives (39, 6.25%), benzene and substituted derivatives (35, 5.61%), phenols (17, 2.72%), and flavonoids (4, 0.64%) (**Supplementary Figure 2**).

**Figure 2:**
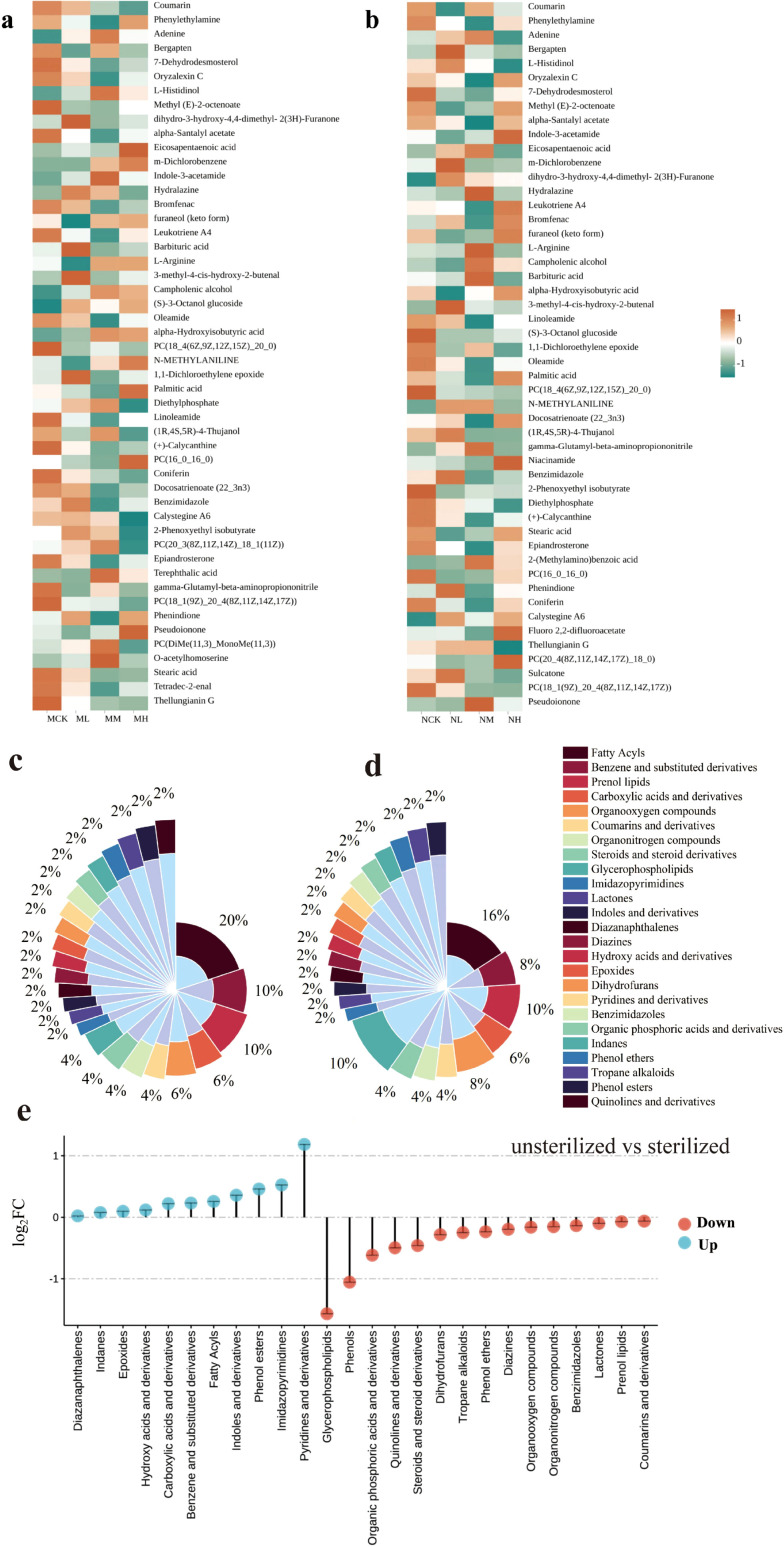
Metabolite analysis of sterilized and unsterilized soil under different concentrations of *Solanum rostratum* Dunal litter treatment. **(a)** Top 50 relative abundances of sterilized soil metabolites; **(b)** Top 50 relative abundances of unsterilized soil metabolites; **(c)** Classification of top 50 relative abundances of unsterilized soil metabolites; **(d)** Classification of top 50 relative abundances of sterilized soil metabolites; **(e)** Changes in the relative abundance of metabolite classes between unsterilized and sterilized soils. The number of soil samples used for statistical analysis is 3).

The top 50 metabolites in both sterilized and unsterilized soils were selected for analysis, as shown in **Figures 2a, b**. Classification of these metabolites (**Figures 2c, d**) revealed that after adding *Solanum rostratum* Dunal litter, compared to sterilized soil, unsterilized soil exhibited a 4% increase in fatty acyls metabolite diversity, along with a 2% rise in benzene and substituted derivatives, pyridines and derivatives, and phenol esters metabolite diversity. While glycerophospholipids metabolites decreased by 6%, and organooxygen compounds and phenols both decreased by 2%. Additionally, we compared the relative concentrations of the top 50 soil metabolites in sterilized and unsterilized soils based on their different classes (**Figure 2e**; **Supplementary Figure 3**). Analysis of the total metabolite sum across low, medium, and high concentration treatments. The results revealed that compared to sterilized soil, unsterilized soil exhibited upregulation in 11 metabolite classes including pyridines and derivatives, imidazopyrimidines, and downregulation in 15 classes including glycerophospholipids, phenols, organic phosphoric acids and derivatives. Notably, in unsterilized soil, the relative abundance of pyridines and derivatives increased by more than twofold, while glycerophospholipids and phenols decreased by more than twofold.

**Figure 3:**
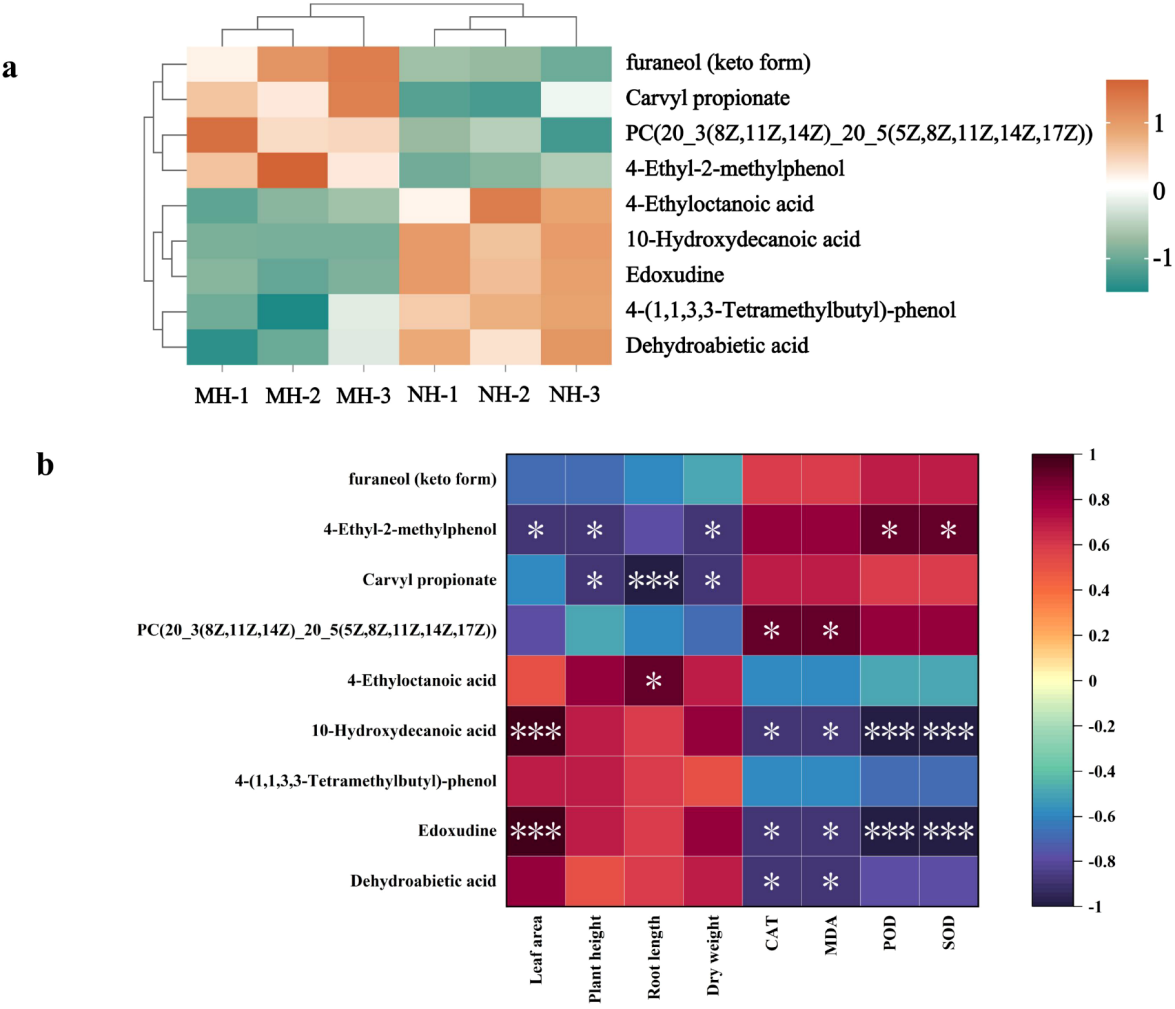
Clustering of differentially expressed metabolites and their correlation analysis with plant physiological indicators. (**(a)** clustering analysis of differentially expressed metabolites; **(b)** correlation analysis of differential metabolites with plant physiological indicators; MH represent sterile conditions: high concentration (20g); NH represent non-sterile conditions: high concentration; Spearman’s rank correlation analysis; *P<0.05, **P<0.01, ***P<0.001. The number of soil samples used for statistical analysis is 3).

After adding high-content *Solanum rostratum* Dunal litter, nine differential metabolites were identified in sterilized and unsterilized soils (**Figure 3a**). These included phenols, prenol lipids, glycerophospholipids, fatty acyls, hydroxy acids and derivatives, purine nucleotides, and benzene and substituted derivatives. Compared to sterilized soil, unsterilized soil exhibited downregulation of four differential metabolites including furaneol (keto form) and carvyl propionate, while five differential metabolites such as 4-ethyloctanoic acid and 10-hydroxydecanoic acid showed upregulation.

Correlation analysis was conducted between differential metabolites and plant growth indicators (**Figure 3b**). Results indicate that 4-Ethyl-2-methylphenol showed a negative correlation with plant leaf area, plant height, and dry weight. Carvyl propionate exhibited a negative correlation with plant height, root length, and dry weight. 4-Ethyloctanoic acid showed a positive correlation with root length. 10-Hydroxydecanoic acid and Edoxudine exhibited positive correlations with leaf area, while showing negative correlations with CAT, MDA, POD, and SOD.

### 3.3 Effects of different concentrations of *Solanum rostratum* Dunal litter treatment on soil microorganisms

Bacterial and fungal community structure and species composition analyses were conducted on unsterilized soil (Text S1). Compared to the control group (NCK), the high-concentration litter group (NH) exhibited a higher number of bacterial unique OTUs and fewer fungal unique OTUs (**Supplementary Figure 4**). Additionally, β-diversity analyses were performed on both bacterial and fungal communities (**Supplementary Figure 5**). Results revealed significant differences among NCK, NL, NM, and NH (*P* < 0.05), indicating that soils under different litter concentrations possess distinct bacterial and fungal community structures. Bacterial and fungal α-diversity analysis (**Supplementary Figure 6**) revealed that ACE and Chao1 indices for bacteria significantly increased across all four concentration gradients, while Shannon indices showed no significant effect. For fungi, ACE and Chao1 indices exhibited no significant differences, but Shannon indices significantly decreased.

**Figure 4:**
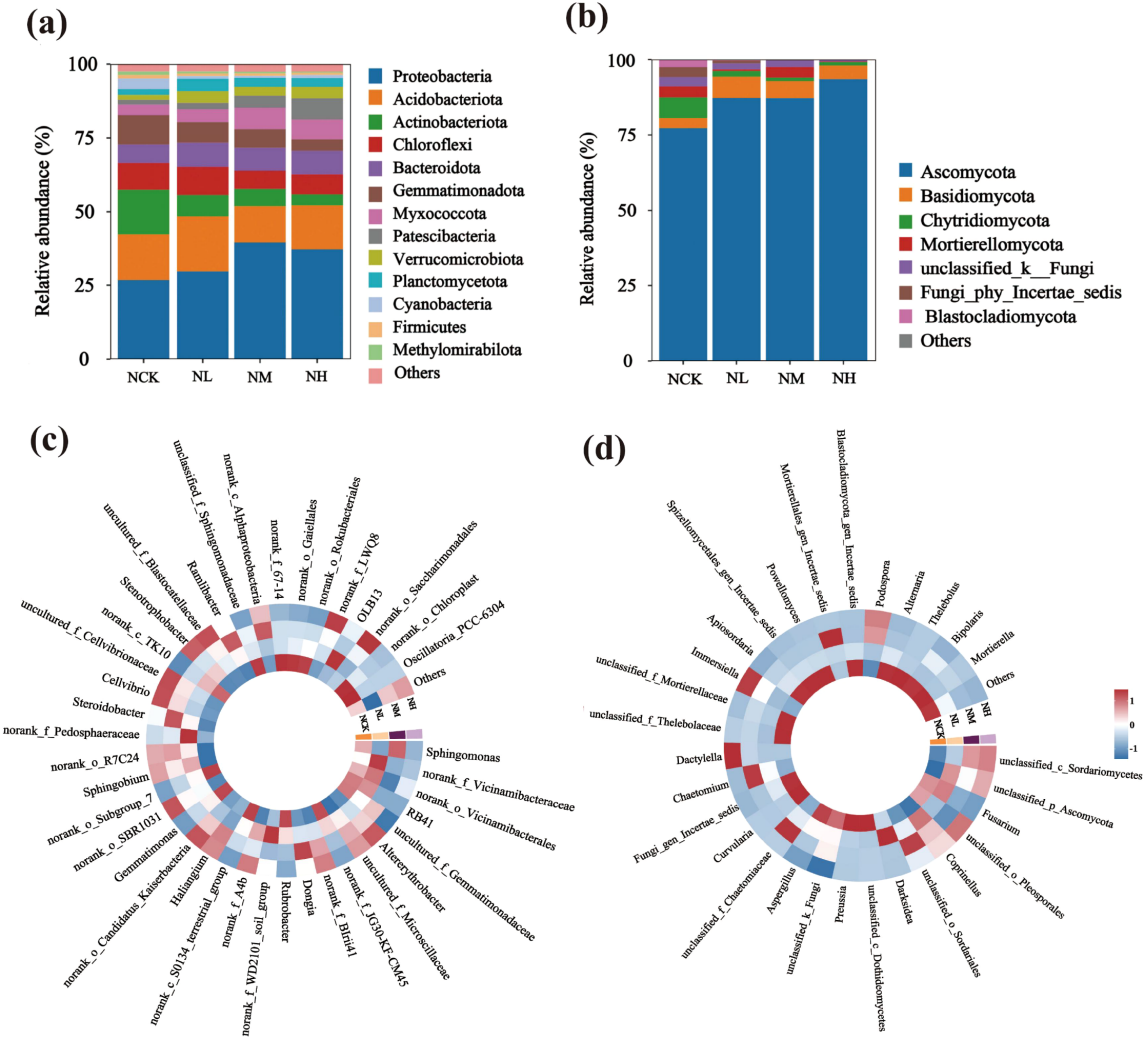
Bacterial and fungal community composition at the phylum and genus levels in unsterilized soil under *Solanum rostratum* Dunal litter treatment. (**(a, c)** bacteria; **(b, d)** fungi; NCK: blank; NL: low concentration; NM: medium concentration; NH: high concentration. The number of soil samples used for statistical analysis is 3).

**Figure 4** shows the community composition of unsterilized soil microorganisms at the phylum and genus levels. Analysis indicates that the dominant bacterial and fungal communities remained unchanged across treatments, though differences existed in relative abundance. At the phylum level, dominant bacterial groups included Proteobacteria, Acidobacteriota, Actinobacteriota, Chloroflexi, Bacteroidota, and Gemmatimonadota (**Figure 4a**). With increasing *Solanum rostratum* Dunal litter content, the relative abundance of Proteobacteria significantly increased (*P* < 0.05), while that of Actinobacteriota and Gemmatimonadota significantly decreased (*P* < 0.05). Compared with the NCK treatment, the relative abundance of Proteobacteria increased by 10.58%, the relative abundance of Actinobacteriota decreased by 11.41%, and the relative abundance of Gemmatimonadota decreased by 6.04% under the NH treatment. The dominant fungal groups in the soil community were Ascomycota, Basidiomycota, and Chytridiomycota (**Figure 4b**). With increasing *Solanum rostratum* Dunal litter content, the relative abundance of Ascomycota significantly increased (*P* < 0.05), while that of Chytridiomycota significantly decreased (*P* < 0.05). Specifically, compared to the control without added *Solanum rostratum Dunal* litter, high-concentration *Solanum rostratum* Dunal litter addition increased the relative abundance of Ascomycota by 16.21% and decreased that of Basidiomycota by 5.69%. At the genus level, the dominant bacterial communities in the soil were *Sphingomonas*, *Altererythrobacter*, *Dongia*, *Rubrobacter*, *Haliangium*, *Gemmatimonas*, and *Sphingobium* (**Figure 4c**). With increasing *Solanum rostratum* Dunal litter content, the relative abundance of *Altererythrobacter* and *Sphingobium* significantly increased (*P* < 0.05), while that of *Rubrobacter* and *Gemmatimonas* significantly decreased (*P* < 0.05). *Altererythrobacter* abundance reached 3.3% under high litter addition levels, compared to 1.4% without litter addition—a 2.4-fold increase. *Sphingobium* abundance increased 2.2-fold under high litter addition levels. *Rubrobacter* abundance was 2.5% without litter addition but decreased to 0.48% with high-concentration litter addition, representing a 5.2-fold reduction. The abundance of *Gemmatimonas* was 1.7% without litter addition and 0.7% with high-concentration litter addition, representing a 2.4-fold decrease. The dominant fungal groups in the soil community were *Fusarium*, *Coprinellus*, *Darksidea*, *Preussia*, *Aspergillus*, and *Curvularia* (**Figure 4d**). Compared to CK, adding high-concentration *Solanum rostratum* Dunal litter significantly increased the relative abundance of *Coprinellus* while significantly decreasing that of *Preussia* and *Aspergillus*. The relative abundance of *Coprinellus* increased from 0.68% to 3.72%, while that of *Preussia* decreased from 7.13% to 0.62%, and *Aspergillus* decreased from 2.90% to 0.17%.

**Figure 5:**
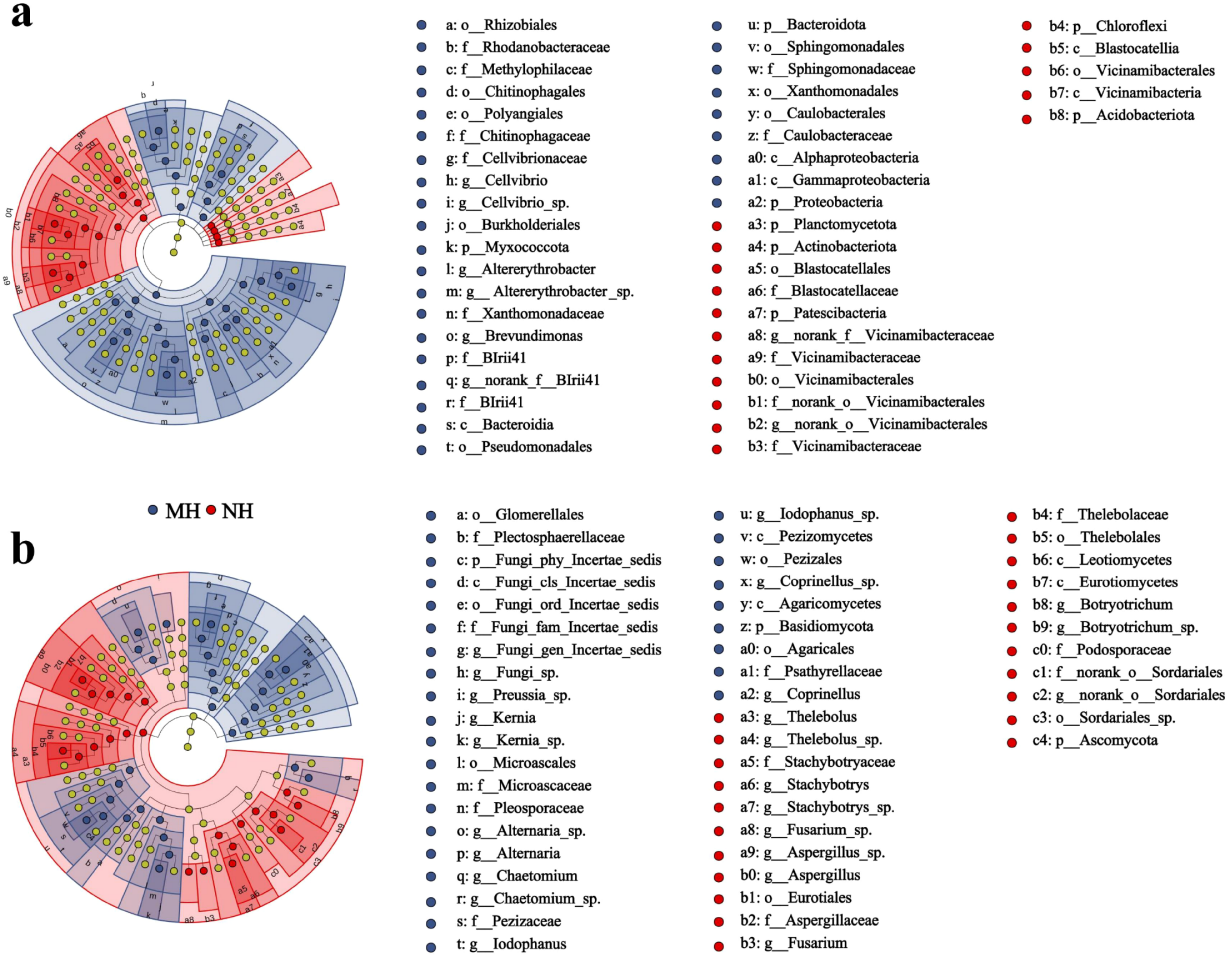
LEfSe analysis under different treatments. (**(a)** bacteria; **(b)** fungi. The number of soil samples used for statistical analysis is 3).

As the dramatic microbial shifts observed specifically under the high-concentration treatment are most directly linked to the phenotypic outcome of interest. So differential biomarkers in high-concentration *Solanum rostratum* Dunal litter addition were identified based on LEfSe analysis (LDA > 3.5) (**Figure 5**, **Supplementary Figure 7**). After comparing high-concentration differential biomarkers with those from soil without added *Solanum rostratum* Dunal litter, 13 characteristic genera were identified at the genus level.

**Figure 6:**
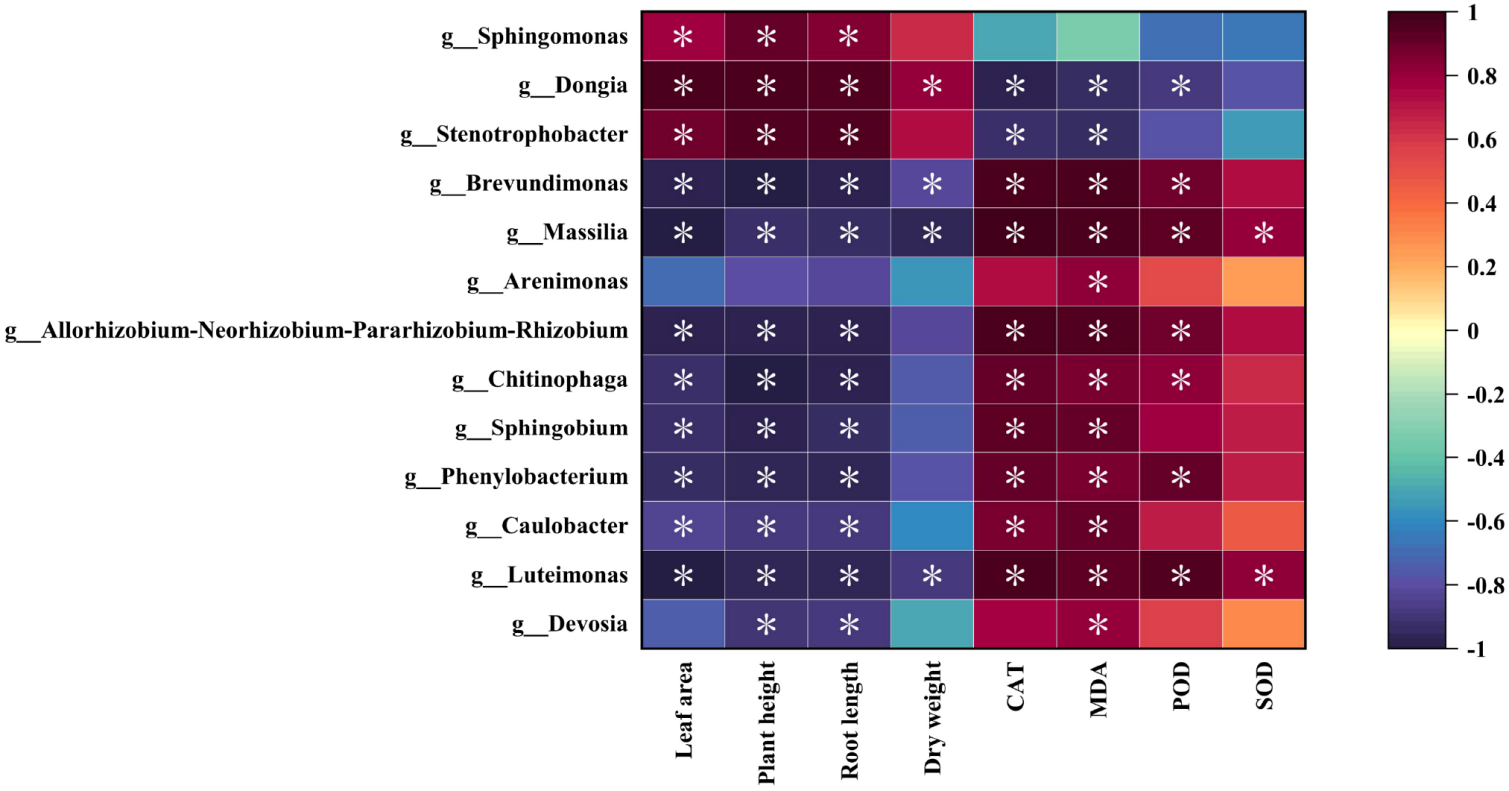
Correlation analysis between differential microorganisms and plant physiological indicators. *P<0.05.

Correlation analysis between differential biomarkers and plant physiological indicators (**Figure 6**) revealed that *Sphingomonas*, *Dongia* and *Stenotrophobacter* showed significant positive correlations with leaf area, plant height, and root length, while exhibiting significant negative correlations with CAT and MDA content. *Dongia* demonstrated a significant positive correlation with biomass, whereas *Dongia* and POD showed significant negative correlations. 10 genera, including *Brevundimonas*a and *Massilia* showed significant negative correlations with leaf area, plant height, and root length, while exhibiting significant positive correlations with CAT, MDA, and POD content.

**Figure 7:**
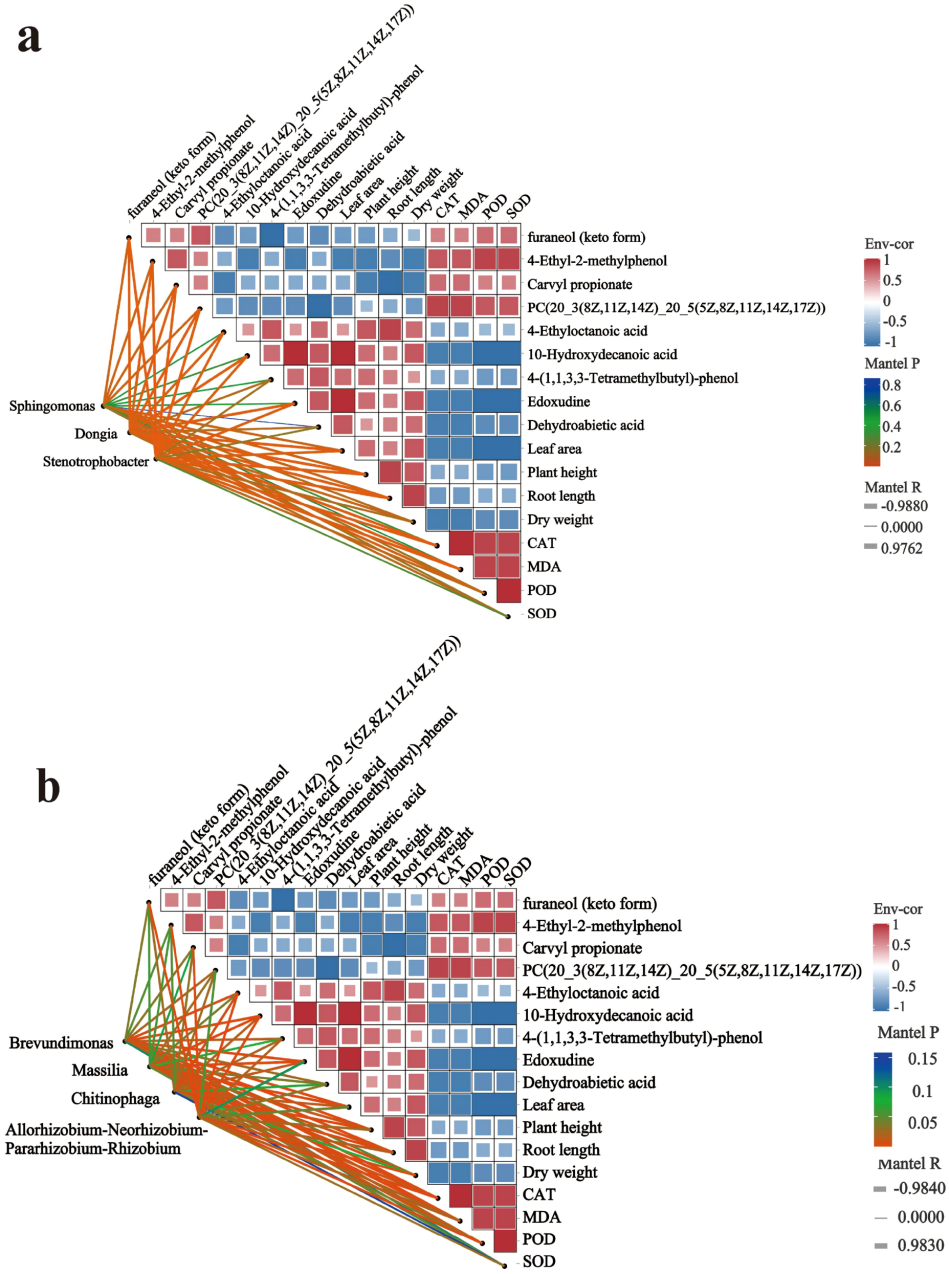
Correlation analysis of differential microorganisms, differential metabolites, and plant physiological indicators (**(a)** positively correlated bacteria; **(b)** negatively correlated bacteria).

Three different microorganisms that were positively correlated with the growth indicators of the seedlings were selected, and their correlations with the differential metabolites were analyzed (**Figure 7a**). Results indicated that *Sphingomonas*, *Dongia*, and *Stenotrophobacter* showed significant negative correlations with metabolites such as furaneol (keto form), 4-Ethyl-2-methylphenol, Carvyl propionate, and PC(20_3(8Z,11Z, 14Z)_20_5(5Z,8Z,11Z,14Z,17Z)) showed significant negative correlations. *Sphingomonas*, *Dongia* and *Stenotrophobacte* exhibited significant positive correlations with leaf area, plant height, root length, and dry weight, while showing significant negative correlations with CAT, MDA, POD, and SOD content.

Similarly, four microorganisms with relatively high abundance and negatively correlated with the growth indicators of the seedlings were selected, and their correlations with the differential metabolites were analyzed (**Figure 7b**). Results indicated that *Brevundimonas, Massilia, Chitinophaga, Allorhizobium-Neorhizobium-Pararhizobium-Rhizobium* showed significant positive correlations with the metabolites furaneol (keto form) and carvyl propionate. *Brevundimonas, Massilia*, and *Chitinophaga* exhibited significant positive correlations with PC(20_3(8Z,11Z,14Z)_20_5(5Z,8Z,11Z,114Z,17Z)). *Allorhizobium-Neorhizobium-Pararhizobium-Rhizobium* exhibited significant positive correlations with 4-Ethyl-2-methylphenol. *Brevundimonas, Massilia, Chitinophaga*, and *Allorhizobium-Neorhizobium-Pararhizobium-Rhizobium* exhibited significant negative correlations with metabolites 4-Ethyloctanoic acid, 10-Hydroxydecanoic acid, and 4-(1,1,3,3-Tetramethylbutyl)-phenol; *Brevundimonas*, *Massilia*, and *Chitinophaga* showed significant negative correlations with Edoxudine; *Brevundimonas*, *Massilia*, and *Allorhizobium-Neorhizobium-Pararhizobium-Rhizobium* exhibited significant negative correlations with Dehydroabietic acid.

## 4 Discussion

### 4.1 Physiological responses of seedlings following the addition of *Solanum rostratum* Dunal litter

Our findings indicate that the addition of *Solanum rostratum* Dunal litter significantly inhibited the growth of its own seedlings, with marked reductions in plant height, root length, and biomass. This aligns with results from other studies. The inhibitory effect on invasive plants increases over time, primarily driven by aboveground litter rather than belowground feedback, and exhibits strong suppression mainly against seedling growth (Fang et al., 2019). This effect may stem from the release of allelochemicals in litter, which directly suppress processes such as energy metabolism, cell division, mineral absorption, and biosynthesis in its seedlings.

Simultaneously, litter accumulation indirectly affects plant growth by altering soil properties. One study found that high concentrations of *Cinnamomum septentrionale* litter suppressed maize morphological traits and pigment content (Yang et al., 2017). Another study demonstrated that litter from the invasive plant Japanese knotweed significantly inhibited the germination and growth of *Leucosinapis alba* (L.) Spach and *Brassica napa* L (Kato-Noguchi, 2021). Similar studies found that *Leucaena leucocephala* (Lam.) de Wit litter inhibited germination and growth in woody plants *Albizia procera* (Roxb.) Benth. and the crop *Vigna unguiculata* (L.) Walp., while increasing mortality rates in five tree species including *Alnus formosana* (Burkill) Makino (Kato-Noguchi and Kurniadie, 2022). Oxidative damage also plays a significant role in allelopathic suppression of plant growth. Following the addition of *Solanum rostratum* Dunal litter in experiments, malondialdehyde, catalase and a series of enzyme activities in seedlings increased. This also indicates that when plants are stressed by allelochemicals, stress-related cellular damage occurs, disrupting redox homeostasis. This leads to the accumulation of reactive oxygen species, causing membrane lipid peroxidation, resulting in a trend of elevated malondialdehyde content. Concurrently, increased reactive oxygen species activate antioxidant enzymes to maintain cellular redox balance. Furthermore, oxidative stress may damage plant mitochondrial structures. Studies have shown that changes in mitochondrial ultrastructure under allelopathic effects inhibit root growth (Šoln et al., 2021, 2022). This aligns with our findings that increased content of *Solanum rostratum* Dunal litter elevated malondialdehyde levels and antioxidant enzyme activity in plants, while simultaneously reducing plant height, root length, and biomass.

We also found that under sterilized conditions, the inhibitory effect of litter was more pronounced. This may be attributed to the fact that unsterilized soil retains its natural microbial community, where certain microorganisms degrade or reduce the toxicity of allelochemicals (Li et al., 2015). Additionally, this may also be related to the associated metabolites. In our experimental results, under non-sterilized conditions, the content of the metabolite 3-Dehydroteasterone was significantly higher than under sterilized. This metabolite, acting as an intermediate in brassinosteroid synthesis (Roh et al., 2020), plays a crucial role in promoting efficient plant growth and enhancing stress resistance (Tanveer et al., 2018; Jiang et al., 2021).

Studies on the physicochemical properties of soil after adding *Solanum rostratum* Dunal litter indicate that the addition of *Solanum rostratum* Dunal litter significantly affected the content of soil nutrients. Soil total carbon, total nitrogen, total phosphorus, soil organic carbon, and available nitrogen contents increased. It also lowered soil pH and increased soil electrical conductivity. This may be related to various metabolites produced during the decomposition of *Solanum rostratum* Dunal litter in the soil, such as cinnamic acid compounds, carboxylic acids and derivatives. The accumulation of these metabolites reduced soil pH and increased soil acidification (Zhang et al., 2025). Previous studies on the effects of invasive species on soil acidity have yielded similar results. Invasive soils colonized by *Fabaceae* and *Rhus typhina* exhibited significantly lower acidity than non-invaded soils (Lazzaro et al., 2014; Wang et al., 2016). Conversely, certain invasive plants showed no significant impact on soil acidity (Stefanowicz et al., 2017; Wang et al., 2017), indicating that the impact of invasive plants on soil acidity varies by species. The increase in soil electrical conductivity may result from the decomposition of litter releasing large amounts of soluble organic matter, cations, and anions (Błońska et al., 2021; Lai et al., 2025). These solutes increase the ionic strength of the soil solution, thereby elevating conductivity. The increase in soil nutrients is closely related to the high organic matter content of the litter itself, as its decomposition directly enhances carbon input into the soil. Furthermore, our results indicate that increasing concentrations of *Solanum rostratum* Dunal litter significantly enhance bacterial diversity in the soil. This, in turn, substantially boosts soil nutrient cycling and increases soil carbon and nitrogen content (Torres et al., 2021).Correlation analysis between soil physicochemical properties and seedling physiological indicators revealed that physiological traits such as plant height and root length exhibited negative correlations with soil nutrient contents including total carbon and total nitrogen. Although the addition of *Solanum rostratum* Dunal litter partially increased soil nutrient levels, the allelopathic effects it induced significantly inhibited plant growth and root nutrient uptake (El-Darier and Zein El-Dien, 2011; Mohammadkhani and Servati, 2018). The increase in soil nutrients cannot offset the inhibitory effect of allelochemicals on plants. In our results, the addition of high-concentration *Solanum rostratum* Dunal litter significantly increased the content of cinnamic acid derivatives in the soil compared to the control without litter addition. Research indicates that cinnamic acid derivatives can inhibit plants’ net nitrate uptake and H^+^-ATP activity in plant plasma membranes (Abenavoli et al., 2010). Some metabolites may interact with nutrients or inhibit plant enzyme activity, thereby negatively impacting nutrient uptake (Chou, 1999). Furthermore, our pH measurements indicate that adding *Solanum rostratum* Dunal litter lowers soil pH. Reduced soil pH also decreases the availability of essential nutrients (Gondal et al., 2021), thereby affecting plant growth.

### 4.2 Significant changes in soil metabolism following the addition of *Solanum rostratum* Dunal litter

Invasive plants typically possess phytochemicals and unique metabolites, which are released into the surrounding environment through mechanisms such as litterfall and rain leaching, thereby influencing plant growth characteristics (Latif et al., 2022; Khamare et al., 2022). Metabolites isolated and identified from soil include fatty acyls, prenol lipids, phenols, flavonoids, and others. These compounds play crucial roles in regulating soil microbial communities, normal plant growth, and defense systems (Kushwaha et al., 2022). Fatty acyls constitute essential components of phospholipids in cell membranes, directly influencing the membrane stability and metabolic activity of soil microorganisms (Borska et al., 2025). Research indicates that prenol lipids, phenols, and flavonoids exhibit allelopathic effects, inhibiting seedling germination and growth (Guo et al., 2023; Kostina-Bednarz et al., 2023). Prenol lipids influence plants to exhibit allelopathic effects through complex interactions involving changes in ATP production, plant endocrine activity, protein complex formation, and respiratory inhibition (Akbar et al., 2024). Furthermore, prenol lipids play critical roles in plant defense mechanisms and responses to abiotic stress challenges (Adams et al., 2023; Khattak et al., 2024). Phenols and flavonoids affect respiratory systems and photosynthetic electron transport chains (Yu et al., 2023). They also inhibit relevant enzyme activities, leading to chlorophyll loss and disrupting carotenoid synthesis, ultimately impairing seedling growth or causing death (Akbar et al., 2024).

In our results, the abundance of pyridines and derivatives metabolites was significantly up-regulated in unsterilized soil compared to sterilized soil, while the abundance of phenols metabolites was down-regulated by more than twofold. In non-sterilized soil, microorganisms can produce pyridines and derivatives by decomposing soil organic matter or through their own metabolism, leading to increased abundance of these metabolites. For example, members of the Proteobacteria phylum can synthesize pyridines and derivatives metabolites via pathways such as polyketide synthase (Popoff et al., 2021). Indeed, our experimental results showed a significant increase in the abundance of the Proteobacteria phylum. Extensive research indicates that phenols metabolites possess strong allelopathic potential (Saludes-Zanfaño et al., 2024; Marchiosi et al., 2020). Their reduced abundance under unsterilized conditions suggests plants may accumulate relevant microorganisms that degrade accumulated phenols compounds in the soil through metabolic pathways, thereby mitigating the toxic effects on their own growth.

Following the addition of high-concentration *Solanum rostratum* Dunal litter, the levels of differential metabolites 4-Ethyl-2-methylphenol and Carvyl propionate decreased significantly in unsterilized soil compared to sterilized soil. Correlation analysis between these differential metabolites and plant physiological indicators revealed a significant negative correlation between both metabolites and plant height, root length, and biomass. 4-Ethyl-2-methylphenol exhibited a significant positive correlation with plant POD and SOD content. Orellana Ávila et al. investigated the inhibitory effects of phenols compounds in allelopathic plants, finding that they suppressed seedling growth by over 50% (Orellana Ávila et al., 2025). Carvyl propionate belongs to the Prenol lipids compounds class. Research has shown that in the presence of this substance, plant seedling growth is significantly inhibited in a concentration-dependent manner (Abd El-Gawad et al., 2016). This is consistent with our experimental findings that under sterile conditions, seedlings exhibited growth abnormalities in the presence of higher concentrations of 4-Ethyl-2-methylphenol and Carvyl propionate.

### 4.3 Changes in soil microbial diversity and community following the addition of *Solanum rostratum* Dunal litter

In this study, the addition of *Solanum rostratum* Dunal litter influenced microbial community structure. Bacterial and fungal α-diversity indices revealed that under different concentration treatments, the ACE and Chao1 indices for soil bacteria significantly increased, while the Shannon index for fungi significantly decreased. This indicates that adding *Solanum rostratum* Dunal litter increased bacterial community species richness while decreasing fungal species diversity. This finding aligns with previous research indicating that litterfall increases bacterial community diversity and abundance. Past studies suggest this may occur because litterfall increases soil nutrients, thereby promoting bacterial growth and diversity (Urbanová et al., 2015). Alternatively, it could stem from endophytic fungi within the litter entering the soil, leading to increased bacterial species richness. Endophytic fungi in litter can alter the abundance and diversity of soil bacterial communities by influencing litter quality and the chemical properties of soil after incorporation (Jin et al., 2022). The decline in fungal diversity may result from allelopathic effects of the litter, allowing more tolerant fungal populations to dominate. Alternatively, it could stem from the rapid growth and heightened activity of soil bacteria suppressing the normal growth of certain fungal populations (Wang and Kuzyakov, 2024; Maan et al., 2022). However, a study on the invasive plant *Reynoutria japonica* revealed that phenolic compounds in its stems and leaves reduced bacterial and fungal biomass, while microbial community functional parameters remained largely unaffected (Stefanowicz et al., 2021). Therefore, the impact of litter on soil microbial diversity may yield different outcomes depending on species differences.

Our analysis of soil microbial community composition revealed that compared to unsterilized soil, the addition of high concentrations of *Solanum rostratum* Dunal litter significantly increased the relative abundance of *Sphingobium* and Ascomycota. This indicates that *Sphingobium* and Ascomycota possess biodegradation and remediation capabilities to respond to stress (Zhao et al., 2017). Studies indicate that plants specifically recruit *Sphingobium* to enhance their own antagonistic activity against pathogens, thereby reducing pathogen infection. This plays a central role in promoting mutualistic relationships between root microbes and plants and maintaining plant health (Li et al., 2023; Wang et al., 2025). Ascomycota represents a key microbial driver, predominantly comprising saprophytic bacteria (He et al., 2022), possessing extensive enzymatic repertoires that confer significant degradation potential (De Vries et al., 2020; Manici et al., 2024). Members of the Ascomycota phylum represent the primary soil fungal decomposers (Vandenkoornhuyse et al., 2002; Bastian et al., 2009), capable of decomposing recalcitrant organic matter in soil. They effectively attack the intermolecular bonds of lignin by producing a wide range of extracellular enzymes (Lynd et al., 2002), thereby promoting nutrient cycling and energy flow (Beimforde et al., 2014). Through the production of diverse extracellular enzymes, they effectively attack intermolecular bonds in lignin (Lynd et al., 2002), thereby promoting nutrient cycling and energy flow (Beimforde et al., 2014).

Correlation analysis between differential biomarkers and plant physiological indicators revealed that *Sphingomonas*, *Dongia* and *Stenotrophobacte* showed significant positive correlations with leaf area, plant height, and root length, while *Dongia* exhibited a positive correlation with biomass. This may be attributed to *Sphingomonas* encoding genes promoting plant growth and root colonization. This bacterial group also possesses genes crucial for auxin and cytokinin biosynthesis (Kämpfer et al., 2025), thereby positively influencing plant traits such as plant height and root length. *Dongia* plays a significant role in nutrient regulation and plant growth (Sanka Loganathachetti and Mundra, 2023). Through its biological nitrogen fixation capability, *Dongia* regulates nutrient allocation to leaves, thereby enhancing plant photosynthetic efficiency and ultimately increasing overall biomass (Xu et al., 2024). This aligns with our experimental findings showing a significant positive correlation between *Dongia* abundance and plant leaf area, plant height, and root length. *Stenotrophobacter* is a bacterium involved in nutrient cycling (Dai et al., 2024; Wang et al., 2022), promoting plant nutrient uptake and thereby stimulating plant growth.

Notably, *Sphingomonas*, *Dongia* and *Stenotrophobacte* exhibited significant positive correlations with plant growth indicators while showing significant negative correlations with the differential metabolites 4-Ethyl-2-methylphenol and Carvyl propionate. Previous studies have indicated that *Sphingomonas* and *Dongia* possess the potential to degrade foreign organisms and complex organic compounds, and also promote plant growth (Guo et al., 2024; Zhang et al., 2020; Ye et al., 2024). This finding indicates that plants may recruit beneficial microbial communities to reduce their own toxic effects and promote plant growth. Additionally, *Brevundimonas, Massilia, Chitinophaga*, and *Allorhizobium-Neorhizobium-Pararhizobium-Rhizobium* exhibited a significant positive correlation with the metabolite carvyl propionate and a significant negative correlation with plant growth. This may be a strategy where plant roots actively enrich certain microbial communities with potential detoxification functions to adapt to stress when growth is inhibited. Previous studies have demonstrated that the enrichment of *Massilia* and *Chitinophaga* can mitigate the effects of stress on plant growth, root colonization, nutrient accumulation, and development (Hong et al., 2024; Fang et al., 2023). Additionally, research indicates that *Brevundimonas* possesses the ability to produce iron carriers, thereby reducing stress responses during adverse conditions (Shang et al., 2025). *Allorhizobium-Neorhizobium-Pararhizobium-Rhizobium* can alleviate continuous cropping obstacles (Chen et al., 2025). However, in our study, despite the increase in relative abundance of the relevant microbial communities, we were unable to successfully reverse the damage inflicted upon the plants. In this study, we simulated the cumulative effects of invasive plant colonization under controlled laboratory conditions. In natural environments, autotoxic compounds from litter do not rapidly accumulate in soil over short periods. Instead, they undergo degradation by soil microorganisms, delaying immediate autotoxic effects. Negative feedback arises only after prolonged colonization (decades) (Li et al., 2015). Future research should therefore investigate and validate the intensity, persistence, and mode of action of autotoxic compounds in *Solanum rostratum* Dunal’s autotoxicity, thereby enhancing our understanding of the specific role autotoxicity plays in its invasion mechanism.

## 5 Conclusion

This study systematically investigated the effects of *Solanum rostratum* Dunal litter on soil microbial community dynamics, soil metabolomics, and seedling growth, while evaluating the role of soil microorganisms in mitigating allelopathic effects induced by its litter. Results indicate that the accumulation of litter of *Solanum rostratum* Dunal forms a concentration gradient in the soil through the continuous release of allelopathic substances, thereby exerting a dose-dependent inhibitory effect on the establishment of invasive plant seedlings. Furthermore, litter addition significantly increased α-diversity in bacterial communities while reducing species diversity in fungal communities, revealing soil microbial community structure responses to litter stimulation. Metabolomic analysis further identified 4-Ethyl-2-methylphenol and Carvyl propionate as key metabolites significantly negatively correlated with plant growth. In unsterilized soil, the abundance of *Sphingomonas* and *Dongia*, possessing degradation potential, increased significantly. Concurrently, the concentrations of 4-Ethyl-2-methylphenol and Carvyl propionate decreased markedly, suggesting that specific soil microorganisms may mitigate the adverse effects of litter allelopathy on plant growth by regulating key metabolite concentrations. In summary, this study demonstrates that soil microorganisms play a crucial regulatory role in mitigating autotoxicity induced by *Solanum rostratum* Dunal litter, particularly through adjusting metabolite dynamics and optimizing soil microbial community structure. This provides new insights into the complex interactions between invasive plants and soil microorganisms. Our research indicates that the self toxicity effect of invasive plants depends on the input of litter and is more regulated by the degradation capacity of soil microorganisms. This reveals a new mechanism by which aboveground litter input and underground microbial processes synergistically regulate population renewal, providing a new dimension for understanding the complexity of intraspecific interactions in invasive plants. Future research requires the isolation and identification of metabolites involved in self toxicity, as well as key microbial groups or functional genes involved in the degradation of self toxic substances.

## Data Availability

The datasets presented in this study can be found in online repositories. The names of the repository/repositories and accession number(s) can be found in the article/**Supplementary Material**.

## Supplementary material

Supplementary Figure 1 The situation of wild invasion of *Solanum rostratum* Dunal.

Supplementary Figure 2 Classification of soil metabolites in potted plants of *Solanum rostratum* Dunal.

Supplementary Figure 3 Changes in relative abundance of metabolite categories between unsterilized and sterilized soils in the blank control group.

Supplementary Figure 4 Number of shared and endemic OTUs of soil bacteria and fungi at different levels of litter concentration.

Supplementary Figure 5 Bacterial and fungal community PCoA based on Bray-Curtis distance.

Supplementary Figure 6 Bacterial and Fungi diversity index.

Supplementary Figure 7 LEfSe analysis under blank conditions.
