# Supplementary material for: The interaction between litter input and soil microbial community regulates the intraspecific allelopathic effects of Solanum rostratum Dunal
Source: Front Plant Sci. 2026 Feb 27;17:1769927. doi: 10.3389/fpls.2026.1769927 (PMC12982403; doi:10.3389/fpls.2026.1769927)
Supplement: Supplementary Figure 1 — The situation of wild invasion of Solanum rostratum Dunal. [file DataSheet1.docx]

***Supporting Information***


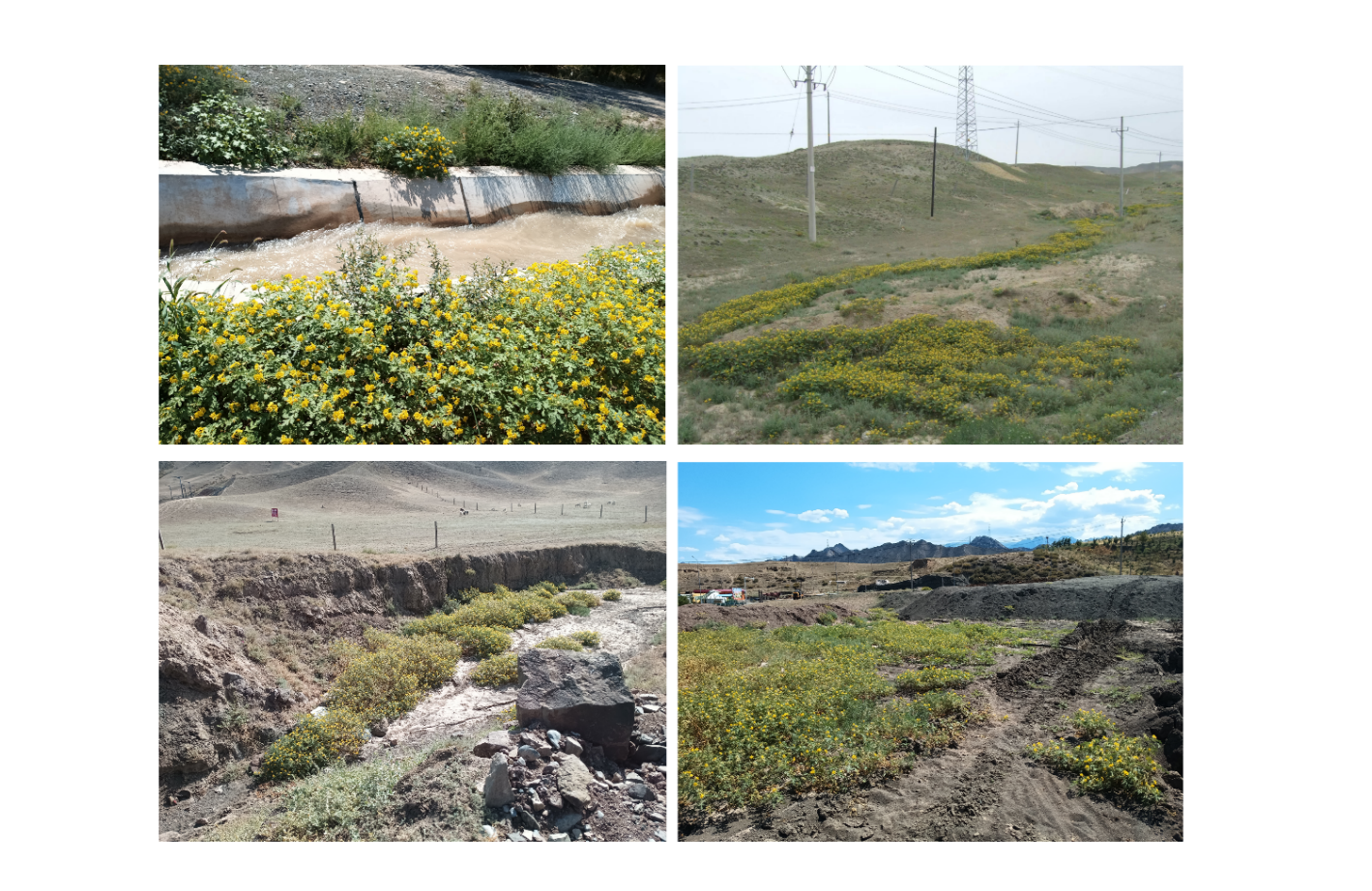


Fig. S1 The situation of wild invasion of *Solanum rostratum* Dunal


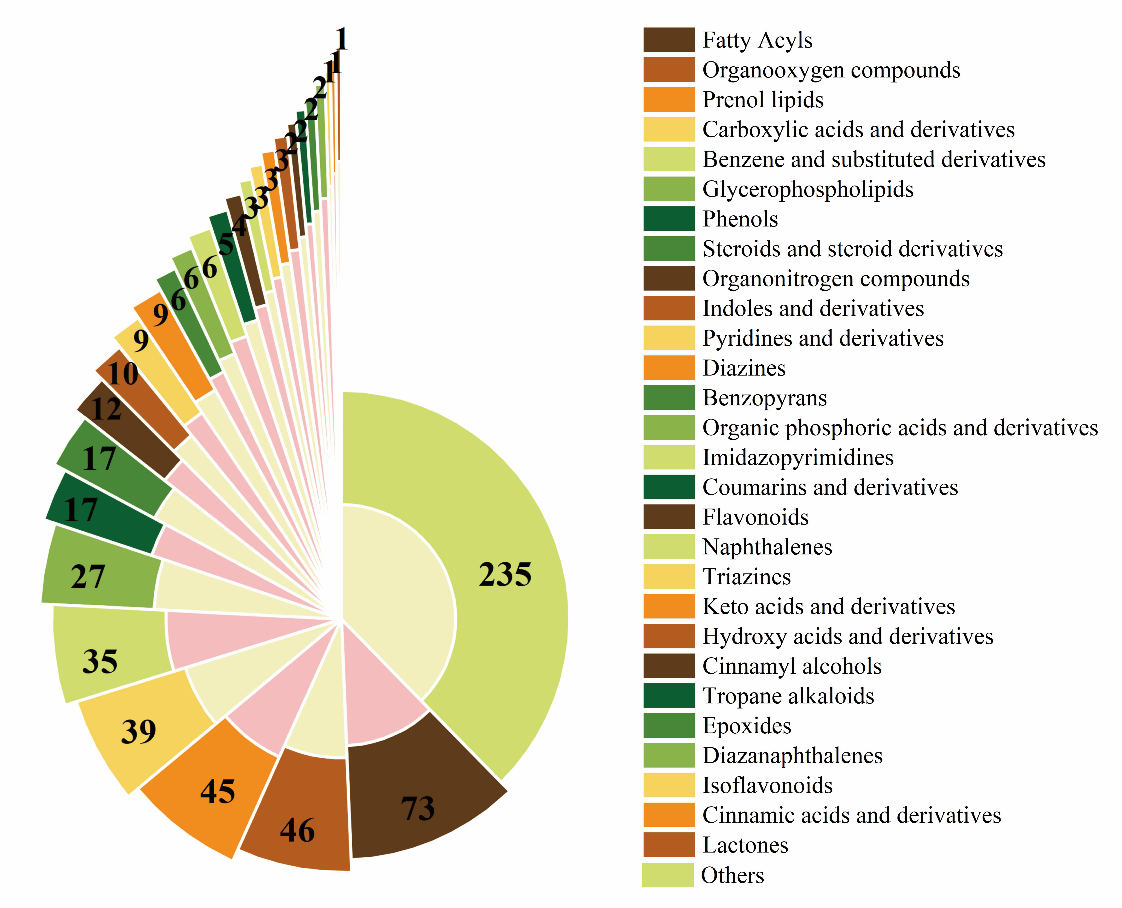


Fig. S2 Classification of soil metabolites in potted plants of *Solanum rostratum* Dunal


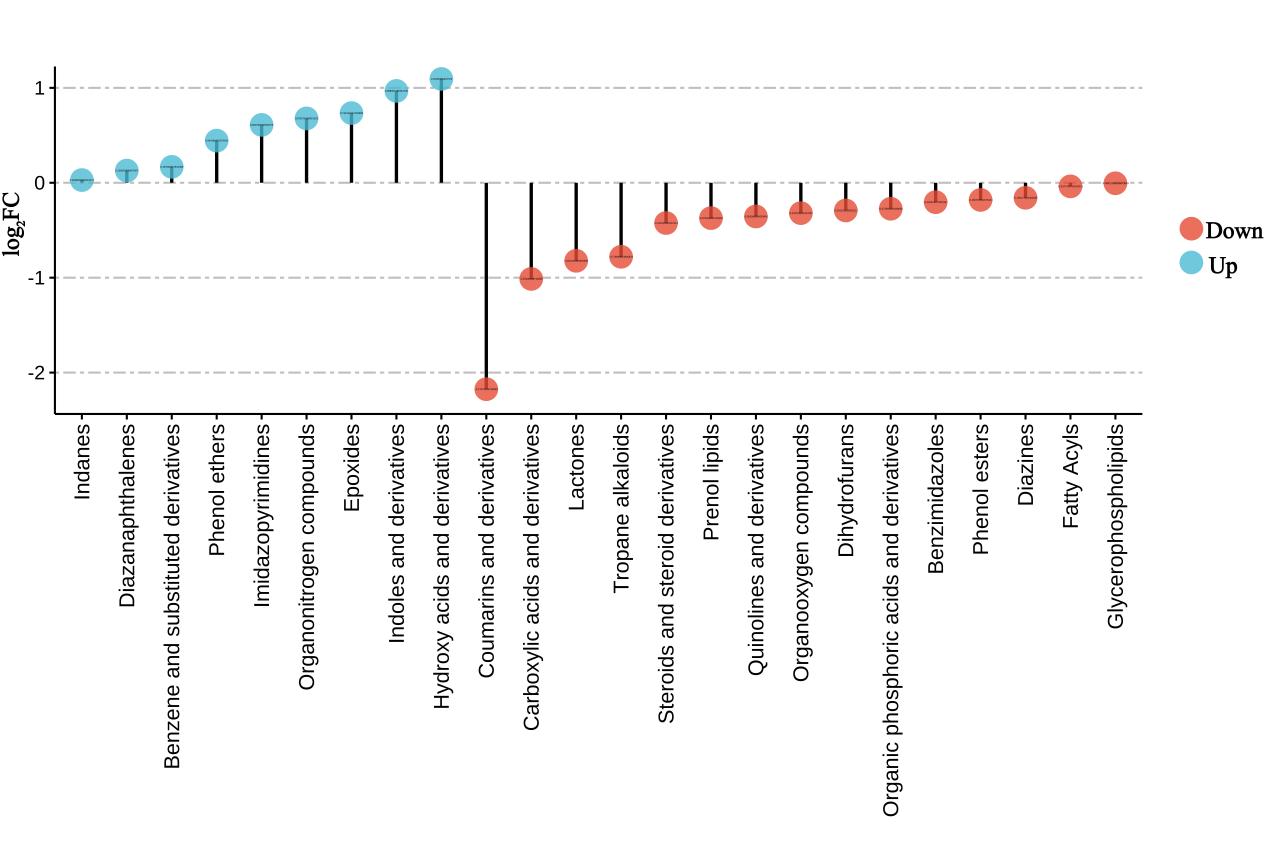


Fig. S3 Changes in relative abundance of metabolite categories between unsterilized and sterilized soils in the blank control group (unsterilized vs sterilized)

**Text S1 Effects of different litter concentrations on the composition and structure of soil bacterial and fungal communities**

The four soils were analyzed for bacterial and fungal community structure and species, and the results revealed that soil bacteria produced a total of 3,129 OTUs and fungi produced a total of 544 OTUs under each concentration of litter treatment, and the common bacterial OTUs and fungal OTUs of the soils under the four treatments were 2,202 and 106, respectively. The high litter concentration group (NH) exhibited a significant increase in bacterial-specific OTUs, while the number of fungal-specific OTUs decreased significantly compared to the no-litter-added control group (NCK) (Fig. S3)


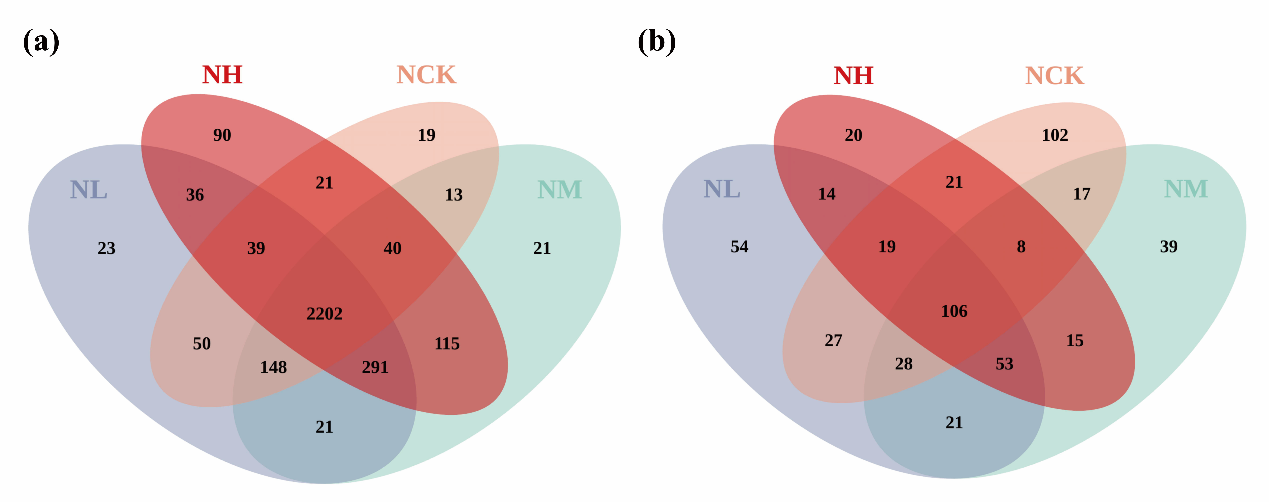


Fig. S4 Number of shared and endemic OTUs of soil bacteria and fungi at different levels of litter concentration (a: bacteria; b: fungi)


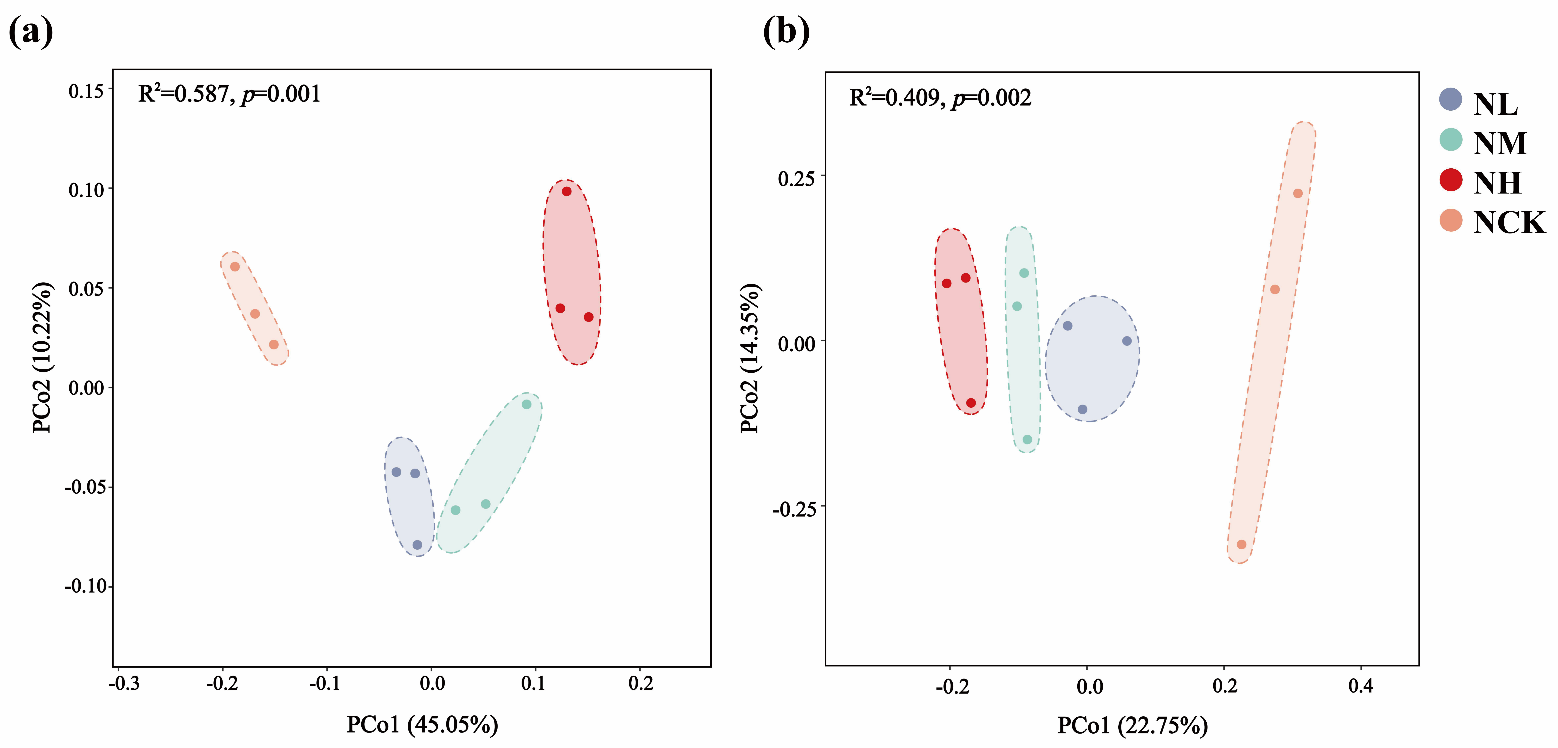


Fig. S5 Bacterial and fungal community PCoA based on Bray-Curtis distance (a: bacteria; b: fungi)


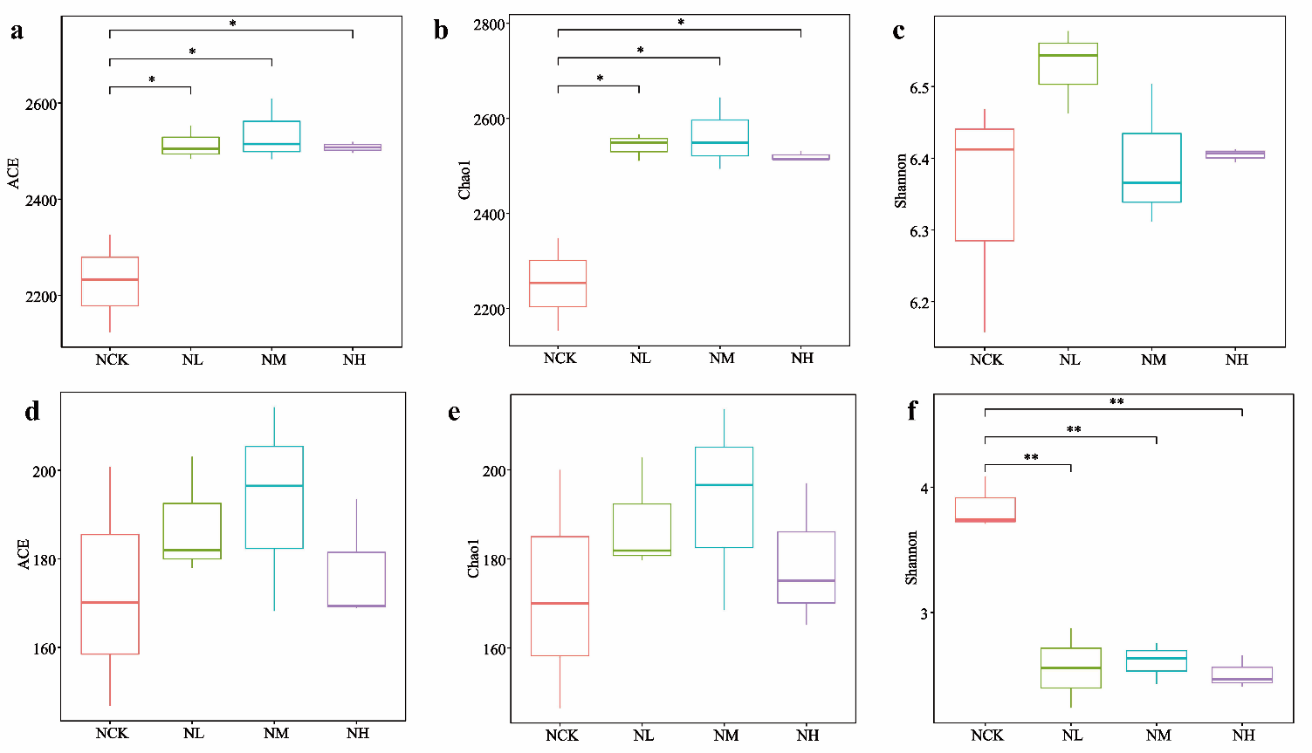


Fig. S6 Bacterial and Fungi diversity index

(a-c: bacteria; d-f: fungi；Wilcoxon; **P*<0.05, ***P*<0.01, ****P*<0.001)


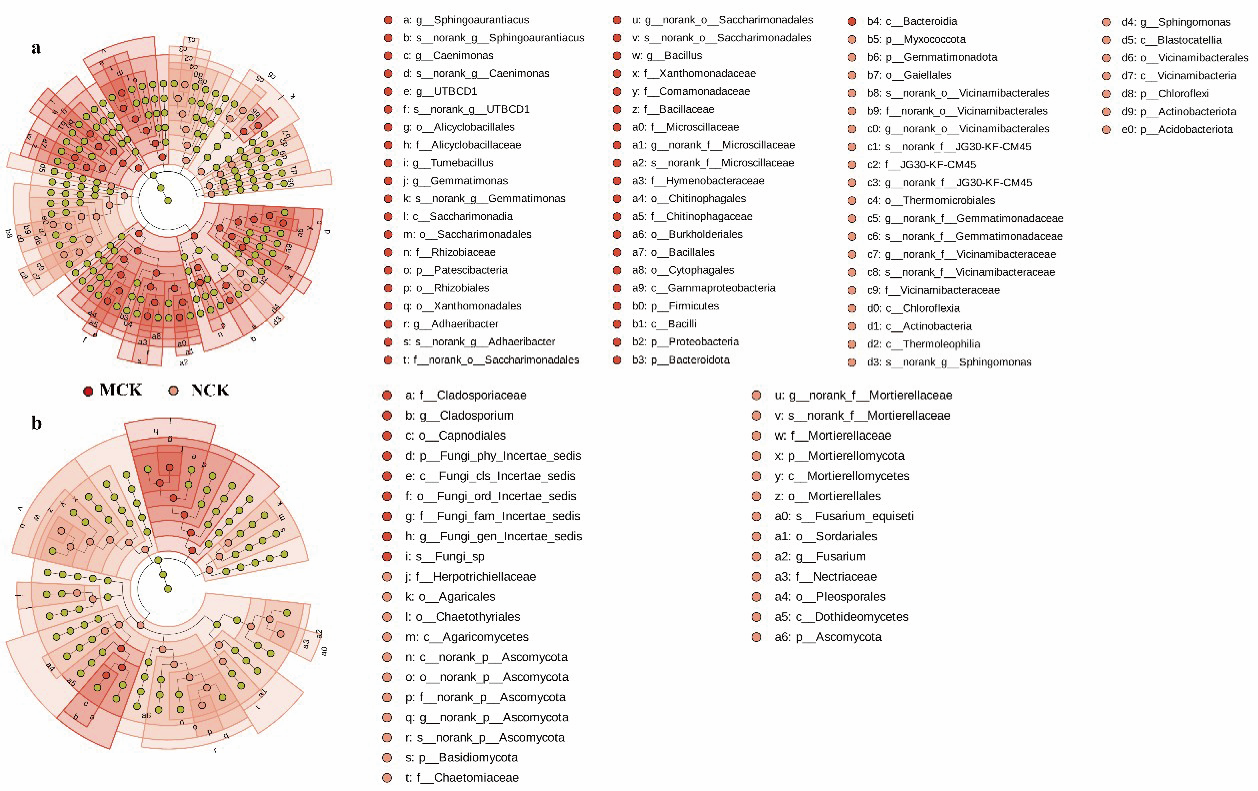


Fig. S7 LEfSe analysis under blank conditions

(a: bacteria; b: fungi. The number of soil samples used for statistical analysis is 3.)
